# Vertebral artery dissection in hypertensive disorders of pregnancy: a case series and literature review

**DOI:** 10.1186/s12884-016-0953-5

**Published:** 2016-07-16

**Authors:** Renuka Shanmugalingam, Nina Reza Pour, Siang Chye Chuah, Thi Mong Vo, Roy Beran, Annemarie Hennessy, Angela Makris

**Affiliations:** Liverpool Hospital, Elizabeth Street, Liverpool, 2170 NSW Australia; Campbelltown Hospital, Therry Road, Campbelltown, 2560 NSW Australia; Western Sydney University, Penrith, Australia; University of New South Wales, Sydney, Australia; Vascular Immunology Research Group, Heart Research Institute, Newtown, Sydney Australia

**Keywords:** Hypertensive disorders of pregnancy, Vertebral artery dissection, Cerebrovascular accident, Hypertension

## Abstract

**Background:**

Arterial dissection is a rare complication of pregnancy and puerperium. There have been reports of aortic, coronary and cervical artery dissection in association with preeclampsia, however, vertebral artery dissection is rarely reported particularly in the antenatal setting in the presence of a Hypertensive Disorder of Pregnancy (HDP).The general annual incidence of symptomatic spontaneous cervicocephalic arterial dissection is 0.0026 % and a data registry reported that 2.4 % of these occurred in the post-partum period. The actual incidence of vertebral artery dissection in HDP is unknown as the current literature consists of case series and reports only with most documenting adverse outcomes. Given the presence of collateral circulation, unilateral vertebral artery dissections may go unrecognised and may be more common than suspected.

**Case presentation:**

We present a case series of four patients with vertebral artery dissection in association with HDP, two of which occurred in the antenatal setting and two in the post-partum setting. All our patients had favourable outcome with no maternal neurological deficit and live infants. Our discussion covers the proposed pathophysiology of vertebral artery dissection in HDP and the management of it.

**Conclusion:**

Our case series highlights the need to consider VAD an important differential diagnosis when assessing pregnant women with headache and neck pain particularly in the context of HDP

## Background

Arterial dissection is a rare complication of pregnancy and puerperium. There have been reports of aortic, coronary and cervical artery dissection in association with pre-eclampsia, however, vertebral artery dissection (VAD) is rarely reported particularly in the antenatal setting in the presence of pre-eclampsia [1, 2]. Four cases of vertebral artery dissection are presented here in the context of hypertensive disorders of pregnancy (HDP). We report two cases of VAD which occurred in the antenatal period and two in the postpartum period. Our case series highlights the importance of recognizing VAD as a differential for occipito-cervical pain in women with HDP.

## Case presentation

### Case 1

**Fig. 1:**
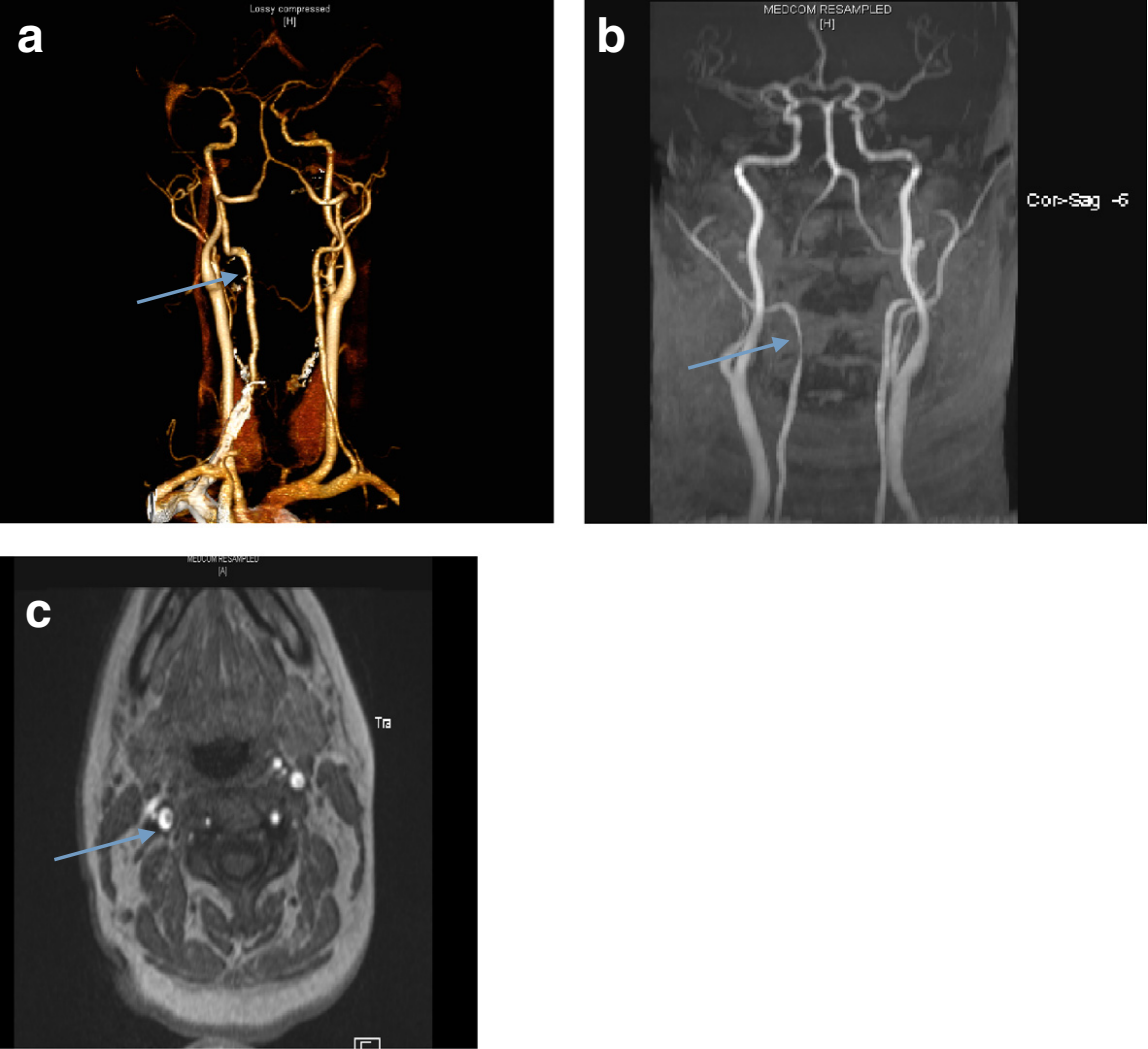
**a** CTA Neck : Focal stenosis with thickened wall and an intramural thrombus of left vertebral artery at the level of C3 (arrow). **b** and **c** A MRI coronal (**b**) and horizontal (**c**) view of the vertebral arteries confirms a 1.1 cm C2-C3 right vertebral artery dissection with no evidence of posterior circulation cerebral infarct (arrow)

A 32 year old primigravid Fijian –Indian non-smoker, with no significant personal or family medical history presented at 38 + 2 weeks gestation in early labour. Her initial blood pressure (BP) was 130/70 mmHg with reassuring cardiotocogram (CTG) trace. She reported a four day history of left neck pain which was described as dull and constant in the post auricular region with radiation down to the base of the neck with no neurological signs on examination. Urine analysis on presentation showed 3+ of protein. Within two hours of presentation, she reported sudden onset of headache and dizziness with a recorded BP of 182/113 mmHg and variable decelerations on CTG. She rapidly progressed on to an eclamptic fit despite oral and intravenous antihypertensive agents. Emergency lower segment caesarean section (LSCS) was performed under general anaesthetic and a male infant (weight 2460 g) was delivered with normal placental histopathology. Retrospective sFlt- 1 (soluble fms-like tyrosine kinase 1) and PlGF (Placental Growth Factor ) concentrations (ELISA, R & D) on serum sample obtained on presentation was 13,150 pg/ml and 190 pg/ml respectively (sFLT-1/PlGF ratio: 69.2). Extubation occurred within 12 h and she reported ongoing neck pain. A computerized tomography angiogram (CTA) of her neck demonstrated a focal stenosis in the left vertebral artery with an intramural thrombus at the level of the third cervical vertebrae (C3) (Fig. 1a). This was further investigated with a magnetic resonance imaging scan (MRI) which confirmed a short segment VAD at C3 with no evidence of posterior circulation cerebral infarct (Fig. 1b and c). She was commenced on aspirin 100 mg daily. A repeat brain and neck CTA at three months post-partum showed resolution of the dissection and aspirin was ceased. A vasculitic screen showed an antinuclear antibody (ANA) titre ratio of 1: 640 with a fine speckled fluorescent distribution. Her double stranded DNA, ENA and lupus anticoagulant were negative. She was initiated on Hydroxychloroquine for possible Systemic Lupus Erythematosis particularly with the onset of polyarthalgia in the postpartum setting.

### Case 2

A 33-year-old primigravid Fijian-Indian non-smoker with no significant personal or family medical history attended a routine antenatal appointment at 36/40 weeks gestation and a BP of 150/110 mmHg was recorded. The spot urinary protein to creatinine ratio (uPCR) was 157.5 mg/mmol and a fetal ultrasound showed appropriate interval fetal growth with no evidence of placental insufficiency. She was initiated on regular oral antihypertensives and admitted for expectant management of pre-eclampsia. Day four of admission (36 + 4/40), she reported rapid onset of right sided neck pain which started at the base of her neck and radiated to the post auricular region. This was concurrent with high diastolic BP readings of 90 - 110 mmHg over 12 h with preserved systolic BP control (125 - 135 mmHg). Dose of regular antihypertensives were increased and an urgent MRI/MRA neck demonstrated a 1.1 cm C2-C3 right vertebral artery dissection with no evidence of posterior circulation cerebral infarct. No inciting event could be recalled. Emergency LSCS was performed and a female infant (weight 2900 g) was delivered with a grossly normal placenta. She was transferred to high dependency unit post-operatively and initiated on a heparin infusion 6 h post-operatively. Within 24 h, she developed progressive abdominal symptoms with haemodynamic compromise necessitating surgical re-exploration and evacuation of haematoma. Heparin was replaced by daily aspirin. A repeat CTA at three months showed resolved dissection and aspirin was ceased. Vasculitic screen was unremarkable.

### Case 3

A 30-year-old Caucasian, gravida two para two, presented six days postpartum with two days history of headache, chest tightness, shortness of breath and bilateral pedal oedema. She was an obese (BMI = 32), non-smoker, with a history of infrequent migraines and no other significant personal or family history. Her first pregnancy, three years prior, was uncomplicated but with different paternity. The antenatal and intrapartum care was uneventful and she delivered a 3800 g male infant at 39 weeks gestation. She was normotensive on discharge, three days later . Day six post-partum, she presented with the symptoms above and reported taking non-steroidal anti-inflammatory drugs (NSAIDs). BP at presentation was 180/110 mmHg and neurological examination was unremarkable. CT pulmonary angiogram (CTPA) and CT Brain (CTB) were both negative for pulmonary embolism and intracerebral haemorrhage respectively. Two days into her admission, she reported ongoing headache with radiation down the right cervical region. Her blood pressure readings ranged between SBP of 130 - 140 mmHg and DBP of 80 -90 mmHg. She underwent a MRI brain which demonstrated an intramural thrombus at the level of C2 indicating a right VAD. Therapeutic dose of subcutaneous low-molecular-weight heparin (LMWH) was commenced in addition to antihypertensive agents. MRA six weeks later demonstrated resolution of the vertebral artery intramural thrombus. LMWH was replaced by aspirin for an additional 6 weeks to complete a total of 3 months of therapy. Antihypertensive medications were ceased as her blood pressure normalized and a vasculitic screen was unremarkable.

### Case 4

A 30-year-old non-smoking Pakistani, gravid 2 para 2, presented at 6 days post-partum with left sided neck pain within 24 h of discharge. Her first pregnancy was complicated by IUGR and post-partum eclampsia. In the second pregnancy, calcium supplement and low dose aspirin were commenced prior to 14 weeks gestation. Antenatal course was unremarkable with the same paternity and a female infant (weight 2720 g) was delivered at 39 weeks gestation. Post-partum period was complicated by post-partum haemorrhage with disseminated intravascular coagulation (DIC) necessitating Ergometrin, intramyometrial Prostaglandin F2 Alpha and transfusion with blood product. She received a single dose of NSAIDs day 3 post-partum, following which her blood pressure was noted to be elevated (maximum 150/98 mmHg). Oral antihypertensive agents were commenced and she was discharged on day 5. She represented within 24 h with a BP of 155/95 mmHg and pain at the left base of her neck with radiation to her ipsilateral shoulder without neurological deficit. A CTA demonstrated a left vertebral artery intramural thrombus at the level of C4/5 for which she was commenced on aspirin 100 mg daily. Her vasculitic screen was unremarkable and a repeat CTA 3 months later showed resolution of thrombus, after which aspirin was ceased.

## Discussion

Vertebral artery dissection is a rare complication of HDP. The general annual incidence of symptomatic spontaneous cervicocephalic arterial dissection (CCAD) is 0.0026 % and a data registry reported that 2.4 % of CCAD occurred in the post-partum period [3, 4]. The actual incidence of VAD in association with HDP is unknown as the current literature consists of case series and reports only with most reporting adverse maternal outcomes (Table 1) [1, 2, 5]. We present two cases of VAD in the antenatal setting and two in the post-partum setting with good maternal and fetal outcomes. VAD is commonly associated with cervical manipulation, rapid head turning or cervical trauma and predisposing conditions includes Marfan’s Syndrome, fibromuscular dysplasia and vasculitis [4]. Our first patient (Case 1) reported having had a neck massage 2 days after the onset of pain. There was no change in the characteristic of her pain following this. There is no known ethnicity predisposition to VAD, therefore, it is unclear if the association between VAD and the ethnicity of our patients (3 of 4 of them were of South - Asian ancestry) is coincidental or due to an underlying genetic predisposition. The common feature in all cases is the presence of a HDP. Two patients (Cases 1 &2) had pre-eclampsia and the remaining two patients (Cases 3&4) had NSAIDs induced post-partum hypertension in an otherwise unremarkable antenatal course. The area of NSAIDs induced hypertension remains subject to ongoing study, though there have been suggestions of NSAIDs precipitating post-partum hypertension in patients at risk [6, 7]. Additionally, the use of Ergometrin and Prostaglandin F2 Alpha, smooth muscle contractors in case 4 could have contributed towards to surge in her previously normal blood pressure.

**Table 1:** Summary of our 4 cases with the case reports in the literature

	Age	Risk factors	HDP	Symptom	Neurological signs	Event time	Treatment	Outcome
Case 1	32	None	Yes - Preeclampsia	Left sided neck pain	No	Antenatal	Aspirin	Alive - no neurological deficit
Case 2	33	None	Yes - preeclampsia	Right sided neck pain	No	Antenatal	Heparin - > Aspirin	Alive - no neurological deficit
Case 3	30	Migraine, Obesity	Yes - NSAID induced postpartum HTN	Headche with left sided neck pain	No	Postnatal	LMWH - > Aspirin	Alive - no neurological deficit
Case 4	30	Previous IUGR and post partum eclampsia	Yes - NSAID induced postpartum HTN	Left sided neck pain	No	Postnatal	Aspirin	Alive - no neurological deficit
McKinney J et al. [1]	41	None	Yes	Headache, HTN, Dysarthria	Quadriplegia	Post natal	Aspirin	Alive with residual memory and visual loss
Arnold M et al. [2]	41	Migraine	- n/a-	Bilateral neck pain	TIA	Post natal	-n/a-	
	35	Migraine, Smoker, HTN	-n/a-	Bilateral headache	Ischemic CVA	Postnatal	-n/a-	Alive, Ischemic CVA
	27	Migraine	-n/a-	Ipsilateral neck pain		Postnatal	-n/a-	
	38	Migraine, HTN	-n/a-	Thunderclap headache		Postnatal	-n/a	
	34	Chiropractor neck manipulation	-n/a-	Ipsilateral neck pain with headache		Postnatal	-n/a-	
Tuluc M et al. [5]	39	None	Yes	Headache preceeding loss of consciousness	LOC	Antenatal	-n/a-	Deceased mother, Live baby
Borelli P et al. [3]	34	None	Yes	Headache with visual disturbance	None	Postnatal	Aspirin	Alive

An imbalance in angiogenesis factors, Placental growth factor (PIGF), soluble FMS-like tyrosine kinase 1(sFlt-1) and soluble endoglin factor (sEng) is present in various degrees of severity in HDPs [8, 9]. It is thought that this biochemical milieu that is well recognized to cause maternal endothelial dysfunction makes the endothelial layer susceptible to intimal rupture [8, 9]. Hypertensive surge is possibly a risk factor for dissections and it is thought that the vertebral artery is prone to the mechanical damage of hypertensive surge given its known vulnerability to traumatic injury [10, 11]. In keeping with that, we observed that the onset of symptoms in our patients was closely related to a preceding surge in blood pressure. We therefore think that the combination of maternal endothelial dysfunction in HDP and the endothelial damaging effect of hypertensive surge potentially increases the risk of dissection in the naturally vulnerable vertebral artery. Passage of blood into this false lumen forces the intimal-medial layer towards the true lumen of the vessel, causing partial or full obstruction of flow, potentially leading to a cerebral ischaemic event [4, 12, 13].

Headache in patients with HDP consist of a broad range of differential diagnosis from benign causes such as migraine to sinister causes such as Posterior Reversible Encephalopathy Syndrome (PRES), Intracerebral haemorrhage, Cerebral venous thrombosis, CCAD and impending eclampsia [14–17]. The incidence of headache in normal pregnancy, however, has been reported to range between 39 % to 59 % hence, creating a clinical dilemma in deciding who should be investigated particularly in the context of HDP [18]. There current recommendation is to investigate headache in pregnant women who have accompanying focal neurology features and those with headache resistant to analgesics though there is no data to support this practice [17, 19]. We investigated our patient in Case 1 for CCAD given the ongoing occipito-cervical pain unrelieved by analgesics. We’ve since had a low threshold in investigating women with HDP who present with headache and neck pain unrelieved by simple analgesics. This led to the identification of VAD in the remaining 3 cases. We suspect that the occurrence of VAD in association with a hypertensive surge in HDP is more common than recognized and should be considered a differential diagnosis when assessing women with headache and neck pain particularly in the context of HDP.

A recent randomised trial showed no difference in efficacy between antiplatelet and anticoagulation in preventing stroke in patients with symptomatic CCAD [20]. There, however, remains a lack of consensus on the need for anticoagulation or antiplatelet altogether as some argue that the risk of secondary cerebrovascular event is low and would not warrant prophylactic therapy. Based on the data and our experience, we, would recommend the use of antiplatelet therapy in the immediate post-partum period given the risk of complications (Case2).

The actual incidence of recurrence of CCAD in subsequent pregnancies is unknown however prophylactic antiplatelet or antithrombotic therapy through the pregnancy would be advisable. There is currently no evidence on the superiority of one over the other in their use prophylactically in this setting. Aspirin has been shown to reduce the risk of pre-eclampsia in high risk women and thus would have a role in subsequent pregnancies of these women – independent of the risk reduction of VAD [21].

## Conclusion

Our case series highlights the need to consider VAD an important differential diagnosis when assessing women with headache and neck pain particularly in the context of HDP. Based on our experience, we would recommend investigating women with HDP who present with head or occipito-cervical pain for VAD particularly in the setting of a hypertensive surge. This is particularly important in the antenatal setting as theoretically, expulsive efforts in labor could risk extending the dissection and therefore VAD in the antenatal setting would warrant a multidisciplinary approach in deciding on a safe mode of delivery [22].

## Abbreviations

ANA, Antinuclear antibody; BMI, Body mass index; BP, Blood Pressure; CTG, Cardiotocography; CCAP, Cervicocephalic arterial dissection;CTA, Computed Tomography Angiography; DIC, Disseminated intravascular coagulation; ENA, Extractable Nuclear Antigen Antibody; HDP, Hypertensive disorders of Pregnancy; IUGR, Intrauterine growth retardation; LMWH, Low Molecular Weight Heparin; LSCS, Lower segment caesarean section; MRI, Magnetic Resonance Imaging; NSAIDs, Nonsteroidal anti-inflammatory drugs; PIGF, Placenta growth factor; PRES, Posterior reversible encephalopathy; s-FLT-1, Soluble fms-like tyrosine kinase - 1;SEng, Soluble endoglin factor; VAD, Vertebral artery dissection
